# The Potential of Artificial Intelligence to Improve Selection Criteria for Liver Transplantation in HCC

**DOI:** 10.3390/cancers17233829

**Published:** 2025-11-29

**Authors:** Jan-Paul Gundlach, Steffen M. Heckl, Patrick Langguth, Christian Oberkofler, Terbish Taivankhuu, Jan Henrik Beckmann, Thomas Becker, Felix Braun, Michael Linecker

**Affiliations:** 1 Department of General, Visceral-, Thoracic-, Transplantation-, and Pediatric Surgery, Campus Kiel, University Hospital Schleswig-Holstein, Arnold-Heller-Street 3, 24105 Kiel, Germanyjan.beckmann@uksh.de (J.H.B.);; 2 Department of Internal Medicine II, University Hospital Schleswig-Holstein, Christian-Albrechts-University, 24105 Kiel, Germany; 3 Department of Radiology and Neuroradiology, Campus Kiel, University Medical Center Schleswig Holstein, 24105 Kiel, Germany; 4 Vivévis Klinik Hirslanden, 8032 Zürich, Switzerland

**Keywords:** HCC, deep learning, liquid biopsy, dynamic selection criteria, liver transplantation, hepatocellular carcinoma

## Abstract

Despite improved therapeutic concepts, the survival of patients with hepatocellular carcinoma (HCC) is limited. Liver transplantation (LT) is the best possible treatment for suitable patients. This therapy is of particular importance, because it not only removes the cancer but also cures the underlying structural liver disease. Due to the persistent lack of donor organs, however, the oncological prognosis after LT is of particular importance for fair organ allocation. Bonus points on the organ waiting list are rewarded for tumors within a certain tumor extent. In general, macrovascular invasion and extrahepatic tumor manifestation are considered to be contraindications for LT, as survival in these patients is very low. In recent years, however, microvascular invasion and poorly differentiated tumors have also turned out to be unfavorable. Most selection criteria for LT in HCC are still based on very simple imaging criteria like size and number without utilizing additional imaging characteristics inherent to the tumor nodule, which could be processed in a “virtual biopsy”. Recently, diagnostic research has presented the clinical benefit of artificial intelligence (AI) in the use of deep-learning strategies for digital diagnosis of poorly differentiated or microvascular-infiltrated tumors. In addition, evaluation of TACE response is analyzed as a possibility to estimate LT survival. The aim of this review is to provide an overview of recent advances in HCC diagnosis and to classify the clinical relevance of these diagnostic and technical advances. Secondly, we discuss how these advances could affect the organ allocation process.

## Simple Summary

Survival in hepatocellular carcinoma (HCC) remains poor despite advances in therapy. Liver transplantation (LT) offers the best curative option, as it removes both the tumor and the underlying liver disease. However, due to the shortage of donor organs, patient selection and oncologic prognosis are crucial for fair organ allocation. While macrovascular invasion and extrahepatic spread are established contraindications, microvascular invasion and poor tumor differentiation have also emerged as negative prognostic factors. Current LT selection criteria mainly rely on simple imaging parameters such as tumor size and number, neglecting deeper imaging features. Recent studies have highlighted the potential of artificial intelligence (AI) and deep-learning methods for detecting aggressive tumor characteristics like microvascular invasion or poor differentiation—offering a “virtual biopsy.” Moreover, assessing tumor response to transarterial chemoembolization (TACE) may help predict post-transplant survival. This review summarizes diagnostic innovations and their potential impact on organ allocation in HCC.

## 1. Introduction

Hepatocellular carcinoma (HCC) remains the fourth leading cancer-related cause of death in both sexes and was the second leading cause of cancer-related death in men worldwide in 2022 [1]. Since the tumor burden of HCC remains high, improved diagnostic and therapeutic strategies are needed to increase overall survival of this devastating disease. Liver transplantation (LT) marks a cornerstone of HCC treatment. In contrast to liver resection, this therapy is of particular importance because it not only removes the cancer but also cures the underlying structural liver disease [2]. Due to the persistent lack of donor organs, however, the oncological prognosis after LT is of immense importance to achieve the best outcomes and ensure fair organ allocation. Bonus points on the organ waiting list are rewarded for tumors within certain tumor margins, such as the Milan criteria (MC). In general, macrovascular invasion and extrahepatic tumor manifestation are considered contraindications for LT worldwide [3]. While, in recent years, microvascular invasion and poorly differentiated tumors have also turned out to be unfavorable [4], most selection criteria for LT in HCC are still based on rigid imaging criteria such as tumor size and number of lesions. Although the accuracy and validity of needle biopsies continues to be debated [5], possible imaging characteristics could fill this gap. Recently, diagnostic research has presented the clinical benefit of artificial intelligence (AI) in the use of deep-learning strategies for digital diagnosis of poorly differentiated [6,7] or microvascular infiltrated tumors [8]. Such modalities could bear the possibility of ‘virtual biopsies’. The aim of this review is twofold: We first provide an overview of previous HCC classification systems from static, image-morphologically focused and histologically oriented characteristics to biological and combined dynamic methods and discuss which tumor size outside of MC represents the upper limit of acceptable tumor recurrence after LT (Part A). Second, recent advances in artificial intelligence-based HCC imaging are reviewed (Part B). Finally, the possibilities of these advances to facilitate precise and non-invasive selection of prognostic groups for LT are summarized.

## 2. Different HCC Classification Systems and Prediction of Tumor Recurrence

### 2.1. Imaging Methods in the Diagnosis of HCC and LI-RADS-Classification

Diagnostic modalities such as ultrasonography (US), computed tomography (CT) and magnetic resonance imaging (MRI) are widely available and generally recommended for detection, staging and surveillance of HCC [9,10]. In the mid and late 1980s, detection of early HCCs was mainly performed by US [11] accompanied by alpha fetoprotein (AFP) screening [12]. Reliable diagnosis based on image-morphological criteria without the need for a tumor biopsy was established in the late 1990s [13,14,15] and based on the characteristic findings from two different imaging methods (contrast-enhanced US, CT, or MRI) in suspicious lesions > 2 cm with arterial hypervascularization [9]. The unique blood supply is of great importance in imaging diagnostics of HCC: while the intranodal number of portal vessels decreases, the number of arteries increases, especially via abnormal and unpaired branches of the hepatic artery. This leads to significant characteristics of HCC lesions in contrast-enhanced imaging, like early contrast uptake in the arterial phase (arterial hyperenhancement) and the so-called wash-out through the veins in following phases [16]. The first European guideline for the detection and treatment of HCC was published in 2001 following the European Association for the Study of the Liver (EASL) conference in Barcelona [9]. At this conference, the screening interval for patients at risk was set at 6 months [9,17]. Although being regularly performed over 20 years, screening of patients at risk was only proven to be beneficial in 2004 [17]. In a study including 18,816 people with a history of hepatitis B or chronic hepatitis, the screening group had an AFP test and US examination every 6 months. In this group, HCC mortality was reduced by 37% compared to the non-screened control group [17].

Advances in imaging have led to improved HCC diagnosis. Techniques such as microbubble-enhanced US [18] as well as perfusion and dual-energy CT [19] have extended the diagnostic horizon. A significant diagnostic advance was achieved through the use of new MRI sequences, including diffusion weighted MRI [20], and hepatobiliary contrast agents such as gadoxetic acid [21]. Classification of suspect nodules is performed according to the protocols of the Liver Imaging-Reporting and Data System (LI-RADS), which is an initiative supported by the American College of Radiology. The LI-RADS protocol was introduced in 2011 and thereafter updated repeatedly [22,23,24]. However, precise diagnosis remains challenging in different circumstances such as small nodules of <3 cm [25] or Metabolic Dysfunction-associated Steatohepatitis (MASH) [26]. In MASH, the diagnosis is more difficult due to a reduced portal wash-out. Although there is evidence that LI-RADS is applicable in MASH [27], the LI-RADS classification is not recommended in MASH so far [26]. A similar recommendation applies for LI-RADS classification in patients with TIPS (transjugular intrahepatic portosystemic shunt), although reports suggest unreserved application in patients with TIPS [28]. In addition, fibrosis grade F4 as well as HCC/cholangiocellular carcinoma (CCC) mixed tumors and malignant portal vein thrombosis [29] are associated with poor graft survival and need to be identified. The latter can be easily classified by contrast imaging methods such as contrast-enhanced ultrasonography (CEUS). A very thorough contemplation of diagnostic modalities in HCC diagnostics was recently published [26].

### 2.2. Organ Allocation Depending on Static Modalities Such as Radiologic and Histological Staging Criteria

Donor organ shortage limits LT as a therapeutic option. The risk of waiting-list drop-out needs to be balanced against tumor recurrence post-transplant [30], which urgently requires a thorough pre-transplant prediction of possible tumor recurrence after transplantation. Therefore, organ allocation for HCC rewards prioritization points for certain tumor characteristics to reflect patient wait-list mortality. In the US, before listing for LT, a 6-month observation period is required to identify patients within MC with aggressive tumor biology in order to exclude those from bonus point granting [31]. While in Europe tumor extent within the MC is also required to grant bonus points (standard exception), such a 6-month rule is not common practice. There are no uniform guidelines on selection criteria for LT.

Different static selection criteria are regularly used today. The MC (a single HCC lesion ≤ 5 cm or multiple HCC ≤ 3 nodules ≤ 3 cm each without macrovascular invasion at pre-transplant imaging) were introduced in 1996 by Mazzaferro et al. for the treatment of small HCC tumors [32]. However, shortly after the presentation of the MC, innumerable studies were carried out to determine whether LT should be withheld from patients who do not meet these criteria [33]. Similar survival rates after 5 years > 75% were demonstrated for the University of California San Francisco (UCSF) criteria (6.5 cm or ≤3 tumors, each ≤4.5 cm with a total tumor volume ≤ 8 cm) [34]. The current most progressive extension of indication for LT is determined by the up-to-seven criteria, which are characterized as HCC with up to seven nodules and the largest node not exceeding 7 cm in diameter and were based on explant pathology. For this group, the 5-year survival rate was reported to be 71% vs. 48% for patients beyond these criteria [35]. However, it took another 2 years, until 2011, for uniform pre-transplant evaluation criteria to be presented for the LI-RADS classification [22].

Since there is evidence that certain tumor types have a poorer outcome after LT, the priority of rapid organ allocation for patients is in question. This includes, in particular, tumors with poor differentiation [36,37] and, above all, microvascular invasion (MVI) [4,35,37]. So far, neither has contraindications for LT but both demonstrate a severe impact on survival: in cases of co-occurrence, the 5-year survival probability of the patients is halved [35]. After MVI was identified as a negative predictor of survival after transplantation in the up-to-seven criteria introduced by Mazzaferro et al. [35], selection criteria were presented which included histological assessment of the presence of MVI. In the Toronto criteria, DuBay et al. proposed biopsy to rule out poor differentiation: together with aggressive locoregional treatment (LRT), survival rates comparable to MC were achieved irrespective of tumor number or size [38]. However, DuBay presented an AFP value > 400 ng/mL to be associated with poorer disease-free survival [38]. Overall, since the a priori detection of poor differentiation and MVI is difficult, different scores with biological parameters were evaluated to approach this problem.

### 2.3. Estimation of the Risk of Tumor Recurrence Depending on Laboratory Scores and Additional Preoperative Parameters

Serum AFP is the most commonly used biomarker; however, not all HCC secrete AFP, and its sensitivity and specificity were demonstrated to be 41–65% and 80–94% in HCC detection [39]. Nevertheless, different scores include serum AFP: Toso et al. proposed a total tumor volume of ≤115 cm^3^ and AFP serum level < 400 ng/mL to be favorable [40], while within the French AFP score, AFP-levels were ranked in <100 ng/mL (0 points), 100–1000 ng/mL (2 points), and >1000 ng/mL (3 points) and added to different points for tumor number and sizes [41]. The metroticket 2.0 classification represents the sum of the tumor diameter and the number of tumor lesions together with the AFP value [42].

Another marker is represented by des-gamma-carboxy prothrombin (DCP), which is, however, rarely used in routine clinical practice in western countries [43]. In the Kyoto criteria, DCP serum levels ≤ 400 mAU/mL together with up to 10 tumor nodes of ≤5 cm each in diameter were demonstrated to indicate 5-year recurrence rates comparable to MC [44,45]. Of note, these criteria have been validated, just like the Kyushu criteria and the MoRAL score, in a living donor (LDLT) cohort [46]. The MoRAL score incorporates both serum levels: the AFP and the DCP values. In the group of patients exceeding the MC, the combination of AFP and DCP levels represented significantly longer recurrence-free and overall survival for patients with a MoRAL score below the cut-off [47]. In recent years, AFP bound to Lens culinaris agglutinin (AFP-L3), a variant of the well-known biomarker AFP, was presented in different studies, mostly in combination with DCP. In general, AFP-L3 seemed to have higher sensitivity and specificity in predicting HCC recurrence than AFP. While a dual biomarker combination of AFP-L3 together with DCP was reported to strongly predict early recurrence [48], the BALAD and BALAD-2 scores combine those three biomarkers together with albumin and bilirubin [49]. Additional combinations with tumor sizes, such as tumor diameter [50] or tumor volume [51] have been suggested: serum levels of AFP-L3 and DCP along with tumor volume were incorporated into the ADV score [51], which was then validated in an international Asian cohort [52]. While DCP, especially in combination, has predictive potential, its application in Germany is infrequent.

More complex scores were developed in recent years incorporating tumor measurements, laboratory parameters, and additional relevant preoperative parameters such as the RELAPSE score (tumor diameter, maximum AFP value, neutrophil–lymphocyte ratio, micro- and macrovascular invasion, and tumor differentiation). This score was just recently developed in the US and validated in a European cohort with overall and recurrence-free survival rates of 69.8% and 66.7% [53]. The HALT score was developed in 2017. Although not internationally validated, the HALT score proposes additional HCC-relevant pre-transplant variables such as cause of cirrhosis and treatment with locoregional therapy for accurate prediction of post-transplant survival [54]. In addition, the LiTES score demonstrated the benefit of non-HCC related variables for the discrimination of risk of tumor recurrence after LT while integrating non-HCC related pre-transplant variables, especially relevant previous illnesses [55].

This suggests the superiority of integrating variables other than tumor-associated factors into a score for reliable prediction of tumor recurrence. Yet, none of these scores have been implemented in the German directive. Scores like neutrophil–lymphocyte or platelets–lymphocyte ratios reflecting systemic host inflammation and molecular markers such as circulating tumor cells are still rare and not clinically validated. Therefore, these markers are not considered in this overview.

### 2.4. Non-Invasive Detection of Microvascular Invasion Before Liver Transplantation

While tumor biopsies might play an important role in the evaluation of targeted therapies [56], the histopathological graduation of HCC nodules before LT has not been included in the diagnostic routine on a regular basis apart from its role in exclusion from CCC [4]. Its utility has been discussed against the downsides of the risk of bleeding, tumor seeding, and sampling errors in multilocular tumors. Up to now, generally, the largest tumor nodule is biopsied with the possible risk of missing MVI in smaller tumor nodules in cases with multilocular tumors.

Several different groups have addressed the prognostic evaluation of preoperative MVI through the use of imaging modalities [57,58]. MVI is usually a microscopic finding and is described as tumor thrombi in small tumor vessels. Correlation of radiological findings such as multifocal tumor lesions [58,59], large tumor sizes [22], non-smooth tumor margins [13,58,60,61], no or incomplete capsules [62,63,64], and intratumoral arteries [63,64] as well as peritumoral arterial enhancement [58] were reported to be associated with an increased risk of MVI—nevertheless, such imaging findings remain observer-dependent. There are numerous other recommendations, including certain MRI sequences and different contrast agents and doses; in the case of CT, among other things, various reconstructions—performed with equipment using the latest technologies—as well as various contrast agent protocols, have expanded the diagnostic possibilities, e.g., different MRI protocols, such as diffusion weighted imaging, were proposed for a noninvasive diagnosis of MVI with a high predictive value [61,65,66]. Of note, Kuo and colleagues presented molecular profiling in 2007 by correlation of tumor imaging features to corresponding gene expression profiles, so called radiogenomics [67]. They identified distinctive imaging findings (so called ‘traits’) and correlated these to gene expression patterns of HCC. Based on previous work identifying gene expression variation in a group of 91 genes that were associated with microscopic venous invasions comprising genes involved in cell proliferation (CDK4, CDC20, MCM5) and matrix invasion (ADAMTS1, MMP14, SPARCL1), these genes were found to be enriched in two predominant imaging traits (presence of ‘internal arteries’ and absence of ‘hypodense halos’). Interestingly, in 30 patients with HCC, tumors with this combination of imaging traits had a 12-fold increased risk of MVI (*p* = 0.004) [67]. These findings were histologically confirmed in a multicenter study in 2015 with a sensitivity and specificity of 76% and 94%, respectively [68].

### 2.5. Response Rate to Locoregional Procedures

Apart from biological criteria, LRT represent dynamic selection criteria. LRT are routine bridging procedures for patients awaiting LT. Transarterial procedures, ablative therapies (radiofrequency ablation, RFA), and liver resection are equally effective in bridging and downstaging before LT in early-stage tumors, although, the quality of evidence in different studies is very low due to selection bias [69]. However, liver resection remains limited to early-stage Child-Pugh A cirrhosis and the other studies included in the meta-analysis from 2018 demonstrated a high degree of heterogeneity. In cases of small resectable tumors (<2 cm) with underlying Child-Pugh A cirrhosis, resection should be performed to spare rare donor organs [70]. So far, in case of irresectability, patients have been presented for percutaneous or transarterial therapeutic strategies, such as transarterial chemoembolization (TACE) or selective internal radiotherapy (SIRT). The transarterial procedure is now the most commonly used bridging procedure for most of the lesions before LT, with a positive correlation with post-transplant survival [71,72]. This also includes patients with tumor burden beyond the MC, and tumor downstaging can be frequently witnessed. TACE treatment is recommended not only for intermediate-stage HCC, but also for early-stage HCC as a stage-migration strategy and neoadjuvant treatment before LT [72]. Nevertheless, a standardization for treatment modalities such as drug administered, and therapeutic sequence has not been defined so far [71,73,74]. In particular, attention should be paid to synergistic effects in the treatment sequence and a plan for the subsequent therapy should be drawn up.

According to the recent S3 guideline in Germany, TACE should be repeated as long as a response can be demonstrated (e.g., mRECIST) and treatable hypervascularized tumor parts remain [75]. Importantly, it is well known that treatment failure of locoregional therapies is associated with poor survival after LT [37]. Assessing the effectiveness of treatment can increase the possibility of fair distribution when allocating rare donor organs: it is known that tumor progression under locoregional bridging therapy affects overall survival [76]. Therefore, the response rate to locoregional procedures can be a criterion for LT eligibility. The accurate evaluation of the tumor size is of particular importance, especially with regard to tumor lesions, for which bonus points (Milan-In) are awarded. The use of the EASL or mRECIST criteria for the TACE response rate should be used as often as possible [77,78]; however, these criteria have not yet become obligatory. Downstaging showed favorable results in groups beyond MC [79,80,81]. Encouraging results from the UCSF downstaging protocol [79] were updated in 2015 with patients receiving HCC therapy between 2002 and 2012 [82]. Patients downstaged (*n* = 118) to within UNOS T2 criteria (*n* = 488) showed comparable 5-year post-transplant survival and recurrence-free results with the UNOS T2 group (77.8% vs. 81% and 90.8% vs. 88%; *p* = 0.69 and *p* = 0.66).

In a retrospective analysis of the United Network for Organ Sharing (UNOS) database, downstaging (UNOS-DS) as a prognostic marker was evaluated [83]. All groups (Milan-In, UNOS-downstaging, and above UNOS during waiting time) received MELD exceptions for HCC. UNOS-DS was comparable in 3-year survival rates with the MC group. In downstaging groups, AFP ≥ 100 ng/mL was the only independent predictor of HCC recurrence [83]. Interestingly, death post-LT was increased in groups with short or intermediate waiting time for a donor organ. So far, only one clinical trial (XXL trial [84]) has compared overall and recurrence-free survival after successful downstaging with LRT. The LT group demonstrated superior event-free survival and overall survival compared to the control group [84]. Wait-list dropout risk scores (e.g., the Mehta et al. waitlist-dropout score [30]) should be co-considered with biology (AFP/composite scores/AI) when prioritizing allocation to maximize transplant benefit and equity as well as the effect of regional wait-time as a biological selection filter [83,84].

## 3. Artificial Intelligence in HCC Diagnosis

### 3.1. Automated Diagnosis of HCC Nodules Using Radiomics

Recently, the advance of modern image morphological techniques such as radiomics and deep learning strategies have enabled the automated diagnosis of HCC [85,86,87]. Table 1 gives an overview of the benefits of non-invasive HCC diagnosis and AI integration. Radiomics represents a diagnostic method producing a quantitative description of medical images by facilitating repetitive tasks: through the use of artificial intelligence, textural information is quantified by extracting the spatial distribution of signal intensities and pixel interrelationships; thus, radiomics allows questions to be answered outside of a possible assessment by radiologists. However, this technique requires preparatory work from a radiologist: of utmost importance is the definition of the region of interest (ROI) on the respective images. A detailed guide on the workflow of radiomics is presented by van Timmeren et al. [88]. The technique has been presented numerous times in the recent past. Of note, in HCC diagnosis, the combination of MRI LI-RADS and radiomics achieved a higher specificity and a higher positive predictive value compared to LI-RADS assessment alone in nodules ≤ 3 cm [85]. In the following table we briefly demonstrate the merits of these methods for the accurate determination of MVI and poorly differentiated tumors before surgery.

In a contemporary summary, 22 studies analyzing radiomics in MVI detection were compared and evaluated [89]. The predictive value (AUC) showed a wide range from 0.69 to 0.94 in the test cohorts and the overall methodological quality was criticized. In all, reproducibility is missing [89]. An evaluation of different radiomics methods for the evaluation of MVI was presented in a study by Ni et al. The group evaluated 1044 sets of texture feature parameters with 21 different radiomics models in a cohort of 206 patients [90]. They analyzed different models of dimensionality reduction combined with different modeling methods. All of those methods were able to detect MVI. The dimensionality reduction by the least absolute shrinkage and selection operator (LASSO) combined with the gradient boosting decision tree (GBDT) demonstrated the highest accuracy. Different other studies evaluated its merit in CT- and MRI-scanning [90,91,92,93,94]. So far, only to a small extent are radiomics developed for detection of preoperative tumor grading [95].

Although this technique greatly advances diagnostics in many ways through the use of big data, radiomics has several shortcomings. The main disadvantage of the radiomics approach is that it is very time-consuming and labor-intensive, necessitating a high human input. Furthermore, there is a high observer bias, which must be considered as an intra-observer bias. In addition, there is impaired reproducibility of this vulnerable method [96]. While deep learning approaches try to overcome these limitations, their image segmentation is still in need of generalizability.

### 3.2. Deep Learning Approaches for Detection of LI-RADS 5 Nodes, MVI, and Locoregional Treatment Success

Due to the fact that the diagnosis of HCC can be made purely on the basis of image morphology [22], this tumor entity is of particular importance in the research of novel diagnostic models. Deep learning strategies consider higher-order imaging patterns as compared to radiomic features, which enables higher diagnostic performance. In a study by Wu et al. in 2020, the performance of contrast-enhanced MRI for diagnosis of HCC was introduced [86]. Although still requiring human specification of the tumor area by centering the lesion in a rectangular box, data was then transferred into a convolutional neural network (CNN). Next, the pre-trained AlexNet CNN model used liver MRI data for LI-RADS tumor grade classification. These LI-RADS deep learning methods demonstrated performances comparable to radiologists in differentiation of LI-RADS grade 3 (LR-3) to LR-4/5 nodules [86]. In addition, the methods were also demonstrated in CT [87]. Furthermore, even small, challenging HCC nodules of ≤2 cm were securely identified [97].

Deep learning strategies for detection of MVI have been evaluated repeatedly [8,98] and compared to radiomics with comparable results, despite being less labor intensive [99,100]: In a recent multicenter study, 750 patients from five Chinese centers were enrolled for retrospective training using contrast enhanced CT (*n* = 306) and gadoxetic acid-enhanced MRI (*n* = 329), which were validated in a prospective cohort of *n* = 115 patients. A superior predictive outcome was demonstrated in the MRI cohort (AUC 0.812 vs. 0.736; sensitivity: 70.4% vs. 57.4%, *p* = 0.015; specificity: 80.3% vs. 86.9%, *p* = 0.052) [8]. The combination of deep learning from CT [98] as well as MRI [101] indicated remarkable predictabilities of 0.845 and 0.931, respectively. Of note, ultrasound-guided deep learning strategies using liver contrast enhancement in combination with clinical data were shown to be able to predict MVI in HCC nodules in two different studies with an AUC between 0.80 and 0.86 and a specificity of 78.6–81.0% [102,103].

Implementation of AI strategies in radiological diagnostics can provide an objective assessment of the therapy response. Although AI enables an unbiased evaluation of therapeutic response after TACE, deep learning strategies can already support pre-interventional prediction of TACE effectiveness, thus avoiding unnecessary treatment in cases of TACE refractoriness. The prognostic benefit of deep learning evaluation of the response to TACE therapy has previously been described in several studies for CT [104,105,106] and selected studies for MRI [107,108]. Therefore, the therapy-naive assessment of the potential of TACE therapy success can lead to interdisciplinary discussion of therapeutic strategies other than TACE-bridging and LT with individualized survival benefit; however, the performance status of deep learning strategies still needs to be validated [72].

### 3.3. How to Facilitate Uniform Decision-Making in the Transplant Conference?

The selection criteria for LT should be updated. Some possibilities can be found in Table 2. Due to the multitude of new diagnostic options in predicting tumor recurrence, it must be clarified which methods represent suitable approaches to identify HCC with reduced overall survival after LT. Taking into account the dangers of tumor seeding through biopsy as well as the possibility of an inaccurate histopathological conclusion due to the location of the biopsy needle (e.g., invasive front vs. tumor necrosis), currently available imaging possibilities can offer a faster and potentially more accurate diagnosis compared to previous possibilities. Furthermore, usually only the largest lesion is biopsied, and a possible differentiated examination of several nodules is not possible. In contrast, deep-learning offers a diagnostic tool free of complication and applicable to all tumor lesions (LI-RADs ≥ III). In addition, the continuous learning of the deep-learning approach leads to increased accuracy and can be expanded to other parallel questions in the future. This represents outstanding advantages over the previous static selection criteria, from MC to the up-to-seven criteria. The radiological diagnosis of HCC nodules also allows combination with dynamic criteria such as the response to LRT, AFP values, and other laboratory parameters, so that the highest possible accuracy of the survival prognosis can be achieved and adequate prioritization on the waiting-list can be pursued. Of note, the reduced survival of patients with large tumor volume contrasts with patients who have a long waiting-list life expectancy due to their small tumor burden, such as those with low AFP and complete response to LRT. These patients are expected to have little short-term benefit from a prioritized LT allocation process. For such patients, a reduction or deprivation in priority status is under discussion [109]. So far, due to the organ shortage, new concepts are needed to improve donor organ availability. The following strategies could improve this dilemma in the meantime.

### 3.4. Ab Initio Transplantation or Salvage LT? Living Donor Liver Transplantation for Everyone?

In the ab initio concept, prophylactic LT is suggested for patients whose composite biology (biomarkers + AI-predicted high MVI probability) signals a high recurrence risk after resection at the time of resection [110,111]. However, MVI occurrence strongly contradicts overall survival benefits. Importantly, in cases of resectable tumors with underlying Child-Pugh A cirrhosis, resection should be performed to spare rare donor organs [70]. In contrast, salvage LT has been recommended for patients without risk factors or portal hypertension being within Milan criteria at the time of tumor recurrence [70]. Due to the continuing lack of organs in Germany, a prioritized salvage LT seems sensible here, in which, similar to the matchMELD, bonus points are awarded on the basis of the probability of survival. A comprehensive amendment of the allocation guidelines would be desirable for this purpose.

Living donor liver transplantation (LDLT) is beneficial, especially in patients with a high risk of wait-list dropout due to tumor burden (beyond Milan) under deceased donor liver transplantation (DDLT) listing criteria but whose objective biology is favorable (low/intermediate AFP, favorable AFP-L3/DCP or MoRAL profile, and low AI-predicted probability of MVI). (ii) Wait-list dropout risk under deceased donor liver transplantation (DDLT) is high due to tumor kinetics, regional organ scarcity, and (iii) patients who demonstrate repeated and durable responses to LRT yet remain ineligible for timely DDLT because of local allocation policies. Such decisions should be guided by composite prognostic models (e.g., Metroticket 2.0 favorable strata) and anchored to measurable biological markers. These scenarios align with the expanded LDLT selection criteria used in the Kyoto/Kyushu/MoRAL frameworks. 

However, LDLT is limited to the availability of possible donors as well as transplant center capability. While LDLT is rather infrequently performed in Germany, its use is very common in the Middle-East and Asia. Therefore, the patients’ individual benefit relies on the transplant centers’ experience. On the other hand, use of LDLT can result in favorable results without consuming a deceased donor organ, thereby preserving DDLT grafts for non-HCC indications or high-benefit HCC cases.

LDLT offers the potential individual benefit without consuming a deceased donor graft but requires rigorous donor evaluation and explicit consent, given the ethical and safety implications. Nevertheless, due to the donor risk, a standardized informed-consent process is essential, including explicit disclosure of donor risks, alternatives (including DDLT and non-transplant options), and the uncertainty inherent in prediction models. Decisions should be made by multidisciplinary tumor boards with transplant expertise and there should be documented institutional pathways to ensure donor safety and equity in organ allocation.

### 3.5. Limitations of AI in HCC Diagnostics and Organ Allocation

Interpretation of deep learning models is a challenging task and AI models are therefore viewed with considerable controversy. First of all, AI reasoning is not necessarily objective, making it difficult to understand how individual decisions are made. Radiomics frequently require time-consuming manual segmentation, show larger reproducibility concerns (inter/intra-observer variability), and have widely variable AUCs in the literature, whereas deep-learning approaches achieve comparable or better MVI prediction with less manual ROI work and improved scalability, while still requiring external multicenter validation. Next, international multi-center training and validation cohorts are needed for fair and reliable results with the necessity of high-quality datasets without missing or unrepresentative data—however, local data protection policies, algorithmic bias, and lack of transparency can create ethical and legal challenges—especially in international cohorts. In addition, AI usually relies on detection of correlation, not causal relationships, which limits robust predictions in complex real-world settings. While AI has the possibility to enhance fairness in organ allocation, it should not replace human judgment in complex, ambiguous, or most importantly, moral decisions. Its mission is to support, not substitute, expert reasoning.

### 3.6. Practical Implications for Transplant Selection

**Figure 1:**
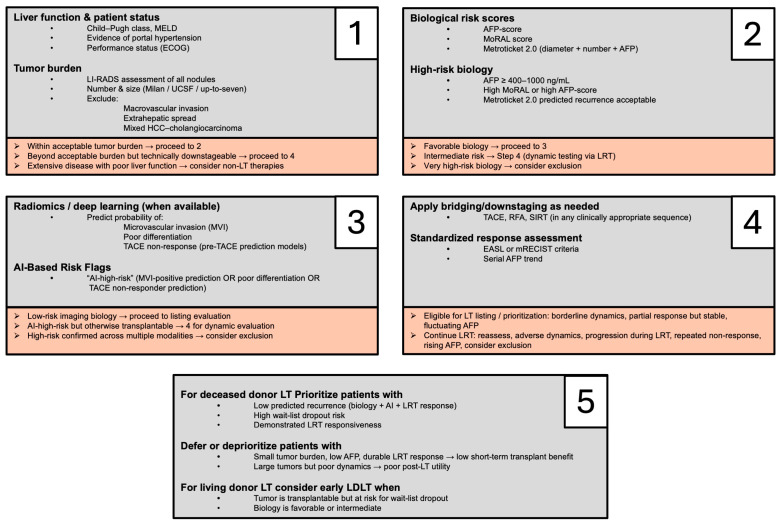
Clinical decision algorithm supporting the use of AI in HCC treatment. This five-step algorithm can easily be used in clinical decision-making for HCC treatment. First of all, liver function, patient status, and tumor burden need to be assessed (**1**). Biological risk scores are subsequently evaluated (**2**). The use of artificial intelligence (AI) can be used for the prediction of microvascular invasion, poorly differentiated tumors, and biological response to locoregional therapy methods (**3**) such as trans-arterial chemoembolization (TACE). In case of bridging to transplant/need of downstaging, locoregional treatment procedures can be used on the basis of guideline recommendations or center’s preference (**4**). Prioritization of liver transplantation is recommended when there is low predicted tumor recurrence probability or high wait-list dropout risk and good response to locoregional treatment procedures (**5**). On the other hand, deprioritization of patients should be considered in case of an expected low short-term transplant benefit. In cases of transplantable tumors with a high risk of wait-list dropout, early living donor liver transplantation (LDLT) should be considered, especially when a possible donor is present, and predicted post-transplant outcomes are expected to match or exceed those achievable through deceased donor liver transplantation (DDLT). Abbr: AFP: alpha fetoprotein; AI: artificial intelligence, LDLT: living donor liver transplantation; LRT: locoregional treatment; LT: liver transplantation, MVI: microvascular invasion, RFA: radiofrequency ablation; SIRT: selective internal radiotherapy TACE: transarterial chemoembolization.

The following figure (Figure 1) represents a possible algorithm in therapy decision-making. In detail, basic liver function and patient status evaluation forms the basis of every therapy decision. In case of acceptable tumor burden (tumors within Milan, UCSF, or up-to-seven), biological assessment via AFP-score, MoRAL score, or Metroticket 2.0 should be applied. In particular, high-risk patients are those with an AFP level ≥ 400–1000 g/mL. In cases with a subsequent low-risk AI risk assessment via deep-learning administration of MVI or tumor grading evaluation, transplant evaluation should be pursued. Subsequent dynamic evaluation with the use of LRT should be incorporated through deep-learning methods in cases of high-risk patients for downstaging/bridging to transplant purposes. In these cases, strict use of the EASL or mRECIST criteria for the LRT response rate should be applied: Complete or partial response sustained for ≥3 months together with stable or falling AFP constitutes a favorable threshold (AFP < 100 ng/mL) to proceed to listing/prioritization; progression or repeated non-response triggers continued LRT or reconsideration of candidacy. Wait-list dropout risk scores should be used along with tumor biology. In addition, regional wait-time (≥6 months) can be used as a biological selection filter.

A different decision aid taking morphologic criteria, AFP, composite biomarkers, and predicted MVI and LRT response into account is presented in Table 3.

## 4. Conclusions

Therapeutic algorithms for early and intermediate stage HCC nodules in Child-Pugh A cirrhosis include resection, while a higher grade of cirrhosis favors LT. Percutaneous or transarterial locoregional treatment approaches are the standard of care for bridging or even downstaging prior to LT, with TACE being the predominant therapeutic option. Unfortunately, data on bridging sequences, such as number of TACE repetitions and the combination of different locoregional treatment procedures, are missing. Therefore, the constant re-evaluation of the therapy response must be taken into account, and future studies should address this important issue.

An allocation of bonus points for the higher prioritization of HCC patients compared to patients without a malignant disease seems sensible. However, the restriction of awarding bonus points only for tumors within the MC is questionable against the background of comparable oncological results in patients meeting, for example, the UCSF criteria. Taking into account the shortage of organ donors, organ allocation based on postoperative survival probability makes sense in order to avoid further fueling the donor organ deficit by expanding the LT indications on the one hand and thus limiting the potential of organ allocation for patients without HCC on the other hand. Since patients with poorly differentiated tumors, microvascular invasion, or progression under locoregional bridging therapy have unfavorable outcomes, they should be excluded from liver transplantation. The implementation of deep learning algorithms might solve this problem (Table 2). The merit of this AI approach is its reproducibility in tumor differentiation (e.g., MVI) and determining the likelihood of a clinical response to bridging treatments while reducing intra-observer viability, especially in comparison with radiomics approaches. The synergy of non-invasive HCC diagnostics, AI-driven continuous learning, and the incorporation of dynamic clinical and biological data results in a robust, patient-centric approach (Table 4). This integration enhances diagnostic accuracy, enables personalized treatment, and improves survival predictions, ultimately leading to better patient outcomes and more efficient healthcare delivery.

## Figures and Tables

**Table 1:** Benefits of non-invasive HCC diagnosis and AI integration.

Category	Key Benefits	Description
non-invasive diagnosis	reduced risk	Avoids biopsy-related complications such as bleeding, infection, or needle-track seeding.
	patient comfort	Enhances compliance and patient acceptance due to painless and simple procedures.
	early detection	Enables identification of HCC at earlier stages via advanced imaging or biomarker analysis.
	cost-effectiveness	Reduces healthcare costs compared to invasive diagnostics.
deep learning and AI	improved diagnostic recision	AI systems trained on large datasets enhance accuracy and reliability in HCC detection.
	adaptability	Continuous learning allows AI to incorporate new biomarkers and imaging features.
	personalized medicine	Integrates patient-specific data for tailored risk assessment and therapy planning.
	predictive analytics	Identifies complex data patterns to forecast disease progression and survival.

Abbr.: AI: artificial intelligence.

**Table 2:** Integration of dynamic criteria.

Category	Key Benefits	Description
dynamic criteria	response to loco-regional therapy	AI evaluates real-time responses to RFA or TACE, refining prognosis and therapy.
	biological parameter response	tracks biomarker dynamics to adjust survival predictions and treatment efficacy.

Abbr.: AI: artificial intelligence; RFA: radiofrequency ablation; TACE: transarterial chemoembolization.

**Table 3:** Decision aid to guide action along different tumor criteria.

Morphologic Criteria	AFP [ng/mL]	Biomarker Composite (AFP-L3/DCP/MoRAL/Metroticket 2.0)	AI-Predicted MVI	LRT Response (EASL/mRECIST)	Waiting-List Action
Within Milan	<100	Favorable	Low	CR/PR or SD	List
	<100	Favorable	High	CR/PR or SD	Continue LRT
	<100	Unfavorable	High	Any	Continue LRT
	100–399	Favorable	Low	CR/PR	List
	100–399	Favorable	High	CR/PR or SD	Continue LRT
	100–399	Unfavorable	Any	PD	Exclude
	≥400	Favorable	Low	CR/PR	Continue LRT
	≥400	Any Unfavorable	High	Any	Exclude
Within UCSF	<100	Favorable	Low	CR/PR	List
	<100	Favorable	High	CR/PR	Continue LRT
	<100	Unfavorable	Any	SD/PD	Continue LRT
	100–399	Favorable	Low	CR/PR	List/Consider LDLT
	100–399	Favorable	High	CR/PR or SD	Continue LRT
	100–399	Unfavorable	Any	PD	Exclude
	≥400	Favorable	Low	CR/PR	Continue LRT
	≥400	Unfavorable	High	Any	Exclude
Within Up-to-Seven	<100	Favorable	Low	CR/PR	Consider LDLT
	<100	Favorable	High	CR/PR	Continue LRT
	<100	Unfavorable	Any	SD/PD	Continue LRT
	100–399	Favorable	Low	CR/PR	Consider LDLT
	100–399	Favorable	High	CR/PR or SD	Continue LRT
	100–399	Unfavorable	Any	PD	Exclude
	≥400	Favorable	Low	CR/PR	Continue LRT
	≥400	Unfavorable	High	Any	Exclude
Beyond Up-to-Seven	Any	Any	Any	Any	Exclude

Abbr.: CR: complete response; LRT: locoregional therapy; PD: progressive disease; SD: stable disease; UCSF: University of California San Francisco criteria. Any: any kind of response to LRT. Exclude: Exclude patient from LT waiting-list.

**Table 4:** Combined benefits of non-invasive HCC diagnosis and dynamic criteria.

Category	Key Benefits	Description
combined benefits	comprehensive monitoring	Continuous, non-invasive follow-up integrating AI-driven insights.
	timely interventions	Facilitates early and precise treatment adjustments, improving survival and quality of life.
	data-driven decisions	Supports evidence-based clinical and allocation decisions.
	enhanced predictive power	Merges AI analytics with biological dynamics for highly individualized survival estimation and therapy design.

Abbr.: AI: artificial intelligence.

## Abbreviations

The following abbreviations are used in this manuscript:

AFP	alpha fetoprotein
AI	artificial intelligence
CCC	cholangiocellular carcinoma
CEUS	contrast-enhanced ultrasonography
CNN	convolutional neural network
CT	computed tomography
DCP	des-gamma-carboxy prothrombin
EASL	European Association for the Study of the Liver
GBDT	gradient boosting decision tree
HCC	hepatocellular carcinoma
LASSO	least absolute shrinkage and selection operator
LI-RADS	Liver Imaging-Reporting and Data System
LRT	locoregional treatment
LT	liver transplantation
MASH	Metabolic Dysfunction-associated Steatohepatitis
MC	Milan criteria
MRI	magnetic resonance imaging
MVI	microvascular invasion
RFA	radiofrequency ablation
SIRT	selective internal radiotherapy
TACE	transarterial chemoembolization
TIPS	transjugular intrahepatic portosystemic shunt
UCSF	University of California San Francisco
UNOS	United Network for Organ Sharing
US	ultrasonography
